# NOD2, RIP2 and IRF5 Play a Critical Role in the Type I Interferon Response to *Mycobacterium tuberculosis*

**DOI:** 10.1371/journal.ppat.1000500

**Published:** 2009-07-03

**Authors:** Amit K. Pandey, Yibin Yang, Zhaozhao Jiang, Sarah M. Fortune, Francois Coulombe, Marcel A. Behr, Katherine A. Fitzgerald, Christopher M. Sassetti, Michelle A. Kelliher

**Affiliations:** 1 Department of Molecular Genetics and Microbiology, University of Massachusetts Medical School, Worcester, Massachusetts, United States of America; 2 Department of Cancer Biology and the Immunology and Virology Program, University of Massachusetts Medical School, Worcester, Massachusetts, United States of America; 3 Department of Medicine and the Immunology and Virology Program, University of Massachusetts Medical School, Worcester, Massachusetts, United States of America; 4 Department of Immunology and Infectious Diseases, Harvard School of Public Health, Boston, Massachusetts, United States of America; 5 Department of Medicine, McGill University Health Centre, Montreal, Quebec, Canada; Institut Pasteur, France

## Abstract

While the recognition of microbial infection often occurs at the cell surface via Toll-like receptors, the cytosol of the cell is also under surveillance for microbial products that breach the cell membrane. An important outcome of cytosolic recognition is the induction of IFNα and IFNβ, which are critical mediators of immunity against both bacteria and viruses. Like many intracellular pathogens, a significant fraction of the transcriptional response to *Mycobacterium tuberculosis* infection depends on these type I interferons, but the recognition pathways responsible remain elusive. In this work, we demonstrate that intraphagosomal *M. tuberculosis* stimulates the cytosolic Nod2 pathway that responds to bacterial peptidoglycan, and this event requires membrane damage that is actively inflicted by the bacterium. Unexpectedly, this recognition triggers the expression of type I interferons in a Tbk1- and Irf5-dependent manner. This response is only partially impaired by the loss of Irf3 and therefore, differs fundamentally from those stimulated by bacterial DNA, which depend entirely on this transcription factor. This difference appears to result from the unusual peptidoglycan produced by mycobacteria, which we show is a uniquely potent agonist of the Nod2/Rip2/Irf5 pathway. Thus, the Nod2 system is specialized to recognize bacteria that actively perturb host membranes and is remarkably sensitive to mycobacteria, perhaps reflecting the strong evolutionary pressure exerted by these pathogens on the mammalian immune system.

## Author Summary

Bacterial and viral infection stimulates production of several cytokines and chemokines that are thought to protect the host against infection. The bacterial strain known to cause tuberculosis elicits production of type I interferons, yet it was unclear how the bacteria isolated within the cell was capable of stimulating this host response. This study reveals that the bacteria use a specialized system to cause damage to these cellular compartments and release bacterial products that activate intracellular innate immune pathways. In this work, we demonstrate that Nod2, Rip2, Tbk-1, Irf3 and Irf5 proteins cooperate to produce type I interferons. Understanding how these pathways are mediated is likely to aid in the design of more effective tuberculosis vaccines.

## Introduction

*Mycobacterium tuberculosis* (Mtb), the causative agent of human tuberculosis, is an exquisitely adapted obligate human pathogen that is thought to persist within as many as one billion individuals worldwide [1]. This bacterium's ability to survive and replicate inside a modified phagosomal compartment of host macrophages is central to the pathogenesis of this disease [2]. While residing at this site, Mtb is able to persist for decades. However, a robust cell-mediated immune response effectively inhibits bacterial replication in approximately 90% of otherwise healthy individuals, and the infection can be controlled indefinitely. Deficits in this immune response result in progressive bacterial replication, necrosis of infected lung tissue, and spread to other individuals. Thus, like many other pathogens that cause chronic infections, the long-term survival of Mtb, depends on a delicate balance between bacterial virulence and host immunity.

Immunity to tuberculosis depends on both the innate and adaptive responses of the host. Initial recognition of the bacterium is mediated by pattern recognition receptors (PRR) such as Toll-like receptors (TLRs) [3],[4] or nucleotide binding oligomerization domain (NOD)-like receptors (NLRs) [5],[6], both of which recognize conserved microbial structures known as pathogen associated molecular patterns (PAMPs). TLRs monitor the extracellular environment and endosomal compartments, and recognize a variety of microbial components including bacterial lipoprotein, peptidoglycan, CpG DNA, and double- and single-stranded RNA [4]. NLRs constitute a more diverse family of approximately 25 proteins, including the caspase-recruiting domain (CARD)-containing Nod1, Nod2 and NLRCs, the pyrin (PYR) domain-containing NLRPs and the baculovirus-inhibitor-of-apoptosis-repeats (BIRs)-containing NLRBs. Nod1 and Nod2 reside in the cytosol and recognize microbial products in this compartment [7]. While the functions of most NLR's remain undefined, the Nod1 and Nod2 proteins have been shown to respond to bacterial cell wall fragments. The Nod1 protein recognizes a fragment of peptidoglycan (PGN) containing the dipeptide γ-d-glutamyl-*meso*-diaminopimelic acid (iE-DAP) produced by Gram-negative and some Gram-positive bacteria. Nod2 recognizes muramyl dipeptide (MDP) present on most types of PGN [8],[9],[10],[11]. While the recognition of these common forms of peptidoglycan have been extensively studied, bacteria modify their cell walls in a myriad of ways and the effects of these modifications on Nod1/2 recognition are only beginning to be appreciated (reviewed in [12],[13],[14]). For example, *Listeria monocytogenes* removes a common *N*-acetyl moiety from the glucosamine of its peptidoglycan, which renders the cell wall resistant to host lysozyme and thereby inhibits bacterial recognition by Nod1 [15]. In contrast, mycobacteria, replace the *N*-acetyl group of the muramic acid of MDP with a *N*-glycolyl moiety[16],[17], and this modification significantly increases the potency of this compound as a Nod2 agonist (Coulombe, F. and Behr, M.A. unpublished data).

Nod1 and Nod2 functions depend on a downstream signaling component, Rip2, which belongs to a protein family currently consisting of 7 members [18]. Like the prototype Rip1, Rip2 contains an N-terminal serine threonine kinase domain followed by an intermediate region and a C-terminal caspase recruitment domain (CARD). Rip2 has been shown to be essential for cytosolic Nod1/2 signaling, and its overexpression stimulates NF-κB activity and induces apoptosis [19],[20]. We have shown that Rip2 is stably modified with ubiquitin in cells treated with the Nod2 agonist MDP [21]. This modification is required for Nod1-mediated NF-κB activation [22], indicating that stable polyubiquitination is a critical component of this signaling cascade.

Intact Mtb bacilli are recognized by both TLRs and NLRs, which cooperatively respond to infection and synergistically induce NF-κB activation [23]. However, a large fraction of the transcriptional response to Mtb, including many immunologically important proteins, such as the chemokines RANTES and IP-10, and the inducible nitric oxide synthase enzyme NOS2 that is critical for mycobacterial immunity, are induced independently of TLR2/4 and the adapter proteins MyD88, MAL and TRIF. Instead these responses rely on autocrine or paracrine signaling via type I interferons (IFNα/β), which are induced through largely undefined pathways [24].

Despite the ability of cell surface localized TLR4 to trigger IFNα and IFNβ transcription, existing evidence indicates that during genuine bacterial infections, this response instead requires the recognition of bacterial products in the cytosol. This has been most clearly demonstrated for pathogens that replicate in the host cell cytosol, such as *Listeria monocytogenes and Francisella tularensis*. In both cases, the bacterium must disrupt the phagosomal membrane and escape into the cytosol in order to trigger the type I IFN response in resting macrophages [25],[26],[27]. Despite its residence in the phagosome, Mtb still induces rapid and robust IFNα/β transcription, and this response depends on a specialized secretion system of the bacterium, ESX1 [28]. This system has been suggested to contribute to the perturbation of the phagosomal membrane [29],[30],[31], indicating that cytosolic recognition might be critical for IFNα/β responses to diverse bacterial pathogens including Mtb.

The primary pathways leading to IFNα/β induction upon bacterial infection remain obscure. Since transfection of DNA into the cytosol of macrophages can induce a Tbk-1 and Irf3-dependent IFNα/β response similar to that seen upon *L. monocytogenes* infection, bacterial DNA has been implicated as the eliciting stimulus [32]. Two different cytosolic DNA sensors have been identified, DAI [33] and AIM2 [34], but their importance during bacterial infections remains to be demonstrated. While Nod2 recognition of MDP is not absolutely required for IFNα/β production [35], it has been shown to synergize with the cytosolic DNA response and enhance IFN production during both *L. monocytogenes* and Mtb infection [36]. However, Nod2 stimulation alone is thought to be insufficient to induce type I IFN production [36].

In sum, while a large fraction of the macrophage response to Mtb infection depends on type I IFN [24] and therefore is likely to rely on a cytosolic signaling pathway, the bacterial products recognized and the pathways involved remain unknown. We previously found that Mtb infection of macrophages triggers Rip2 polyubiquitination in a TLR and MyD88 independent manner [21]. We now show that this stimulation is due to the ESX1-dependent entry of bacterial products into the cytosol where they are recognized by Nod2, implicating MDP as the relevant PAMP. Unexpectedly, this results in IFNα/β production that is dependent on a novel pathway consisting of Nod2, Rip2, Tbk1, and Irf5. This work is the first to implicate NLRs in IRF activation and to suggest a role for Irf5 in anti-bacterial innate immune responses. Furthermore, we found that the unusual *N*-glycolyl MDP produced by Mtb was 10–100 fold more potent than the commonly studied *N*-acetylated MDP produced by most bacteria, and that only *N*-glycolyl MDP could stimulate Rip2-dependent IFNα/β transcription in the absence of other stimulants. Thus, the mammalian Nod2 pathway appears to be remarkably sensitive to mycobacterial MDP and responds to infection by triggering the production of type I interferon, which is responsible for a significant component of the transcriptional response to Mtb infection.

## Results

### *Mycobacterium tuberculosis* infection stimulates the ubiquitin modification of Rip2 via the Nod2 protein

**Figure 1:**
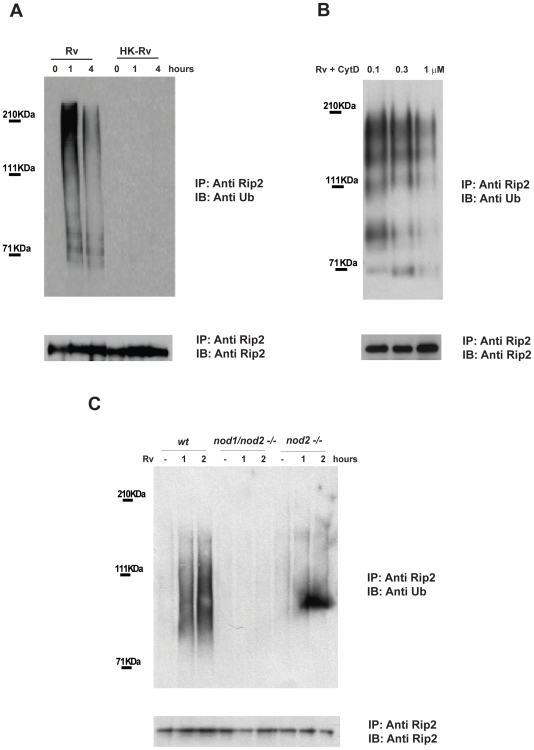
Live, intracellular *Mycobacterium tuberculosis* stimulates Rip2 polyubiquitination. A. Live, but not heat killed Mtb (Rv) stimulates Rip2 polyubiquitination. Bone marrow-derived macrophages (BMDM) were infected with live virulent (Rv) or heat killed (HK-Rv) strains of *M. tuberculosis* (strain H37Rv) for the indicated times. Polyubiquitinated Rip2 protein was detected by immunoprecipitating the cell lysates with an anti-Rip2 antibody followed by immunoblotting with an anti-ubiquitin antibody. Immunoprecipitates were also immunoblotted with a Rip2 antibody (lower panel) to insure that equal amounts of protein were immunoprecipitated. B. The cell permeable mycotoxin Cytochalasin D inhibits Rip2 polyubiquitination upon Mtb infection. The murine RAW 264.7 macrophage cell line was pretreated with cytochalasin D (cytD) for 1 hour at the indicated concentrations before being infected with virulent Mtb H37Rv (Rv) for 1 hour. Polyubiquitinated Rip2 proteins were detected as described above. Treatment with 1 µM cytD caused a 95% decrease in phagocytosis, as described in the Materials and Methods section. C. *Mycobacterium tuberculosis*-induced Rip2 polyubiquitination is Nod2-dependent. Wildtype, *nod1/nod2−/−* or *nod2−/−* bone marrow-derived macrophages cell lines were generated (see Materials and Methods) and either left uninfected (−) or infected with live virulent *M. tuberculosis* (H37Rv) for the indicated times. Polyubiquitinated Rip2 proteins were detected as described above.

The ability of Mtb to rapidly modify macrophage signaling and vesicular sorting pathways [2] suggests that bacterial products gain access to the cytosol soon after phagocytosis. These products are, in turn, likely to be sensed by the host and trigger the innate immune response. Previously, we demonstrated that Mtb rapidly induces the TLR2/4 independent polyubiquitination of the Rip2 protein [21], an event that could represent the initiation of cytosolic recognition. To characterize these events in more detail, we infected the mouse macrophage cell line RAW 264.7 or primary bone marrow derived macrophages (BMDM) with live or heat killed Mtb. In both cell types, we observed that infection with live, but not heat-killed, Mtb stimulated the rapid polyubiquitination of Rip2. The Mtb-induced ubiquitin modification reached maximal levels within 1 hour post-infection and declined by 4 hours (Figure 1A). Furthermore, pretreatment of cells with cytochalasin D to inhibit phagocytosis reduced Rip2 polyubiquitination in a dose-dependent manner (Figure 1B), indicating that the bacteria must be both live and intracellular to initiate this response.

Since Nod1 and Nod2 have been implicated in the cytosolic recognition of mycobacterial components [23], we sought to determine if Rip2 polyubiquitination depended on these proteins. In contrast to cells from wild type mice, inducible Rip2 polyubiquitination was not observed in macrophages derived from mice lacking Nod1 and Nod2, and was greatly reduced in cells lacking only Nod2 (Figure 1C). These data confirmed that intracellular Mtb is recognized by a Nod2-dependent pathway and that this protein is required for the stable ubiquitination of Rip2.

### The mycobacterial ESX1 system is required for Nod recognition

**Figure 2:**
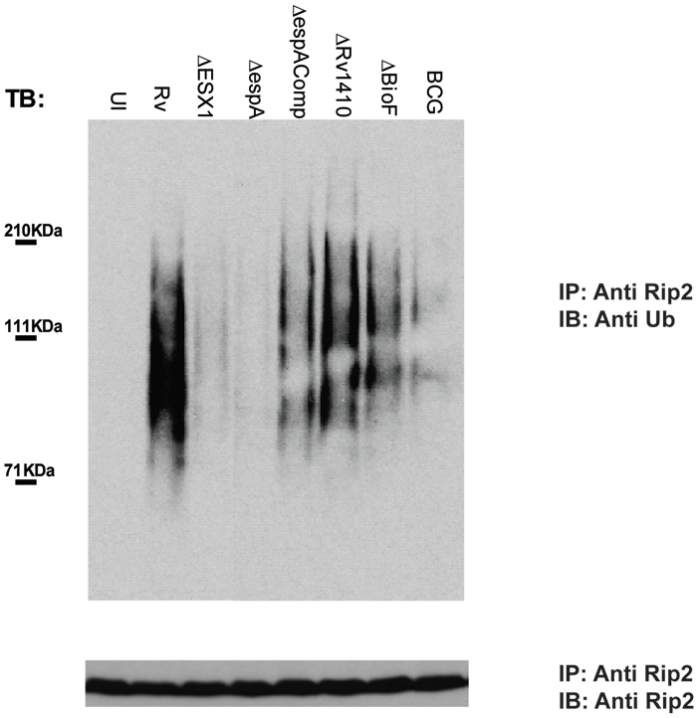
Rip2 polyubiquitination upon Mtb infection requires ESX-1. The murine RAW 264.7 macrophage cell line was left uninfected (UI), infected with Mtb H37Rv (Rv), with ESX1 mutant strains of Mtb (ΔESX1, Δ*espA*), with a complemented Δ*espA*-C strain, with the attenuated vaccine strain *Mycobacterium bovis* BCG or with the unrelated attenuated mutants Tn*::rv1410* and Δ*bioF*. Cell lysates were immunoprecipitated with an anti-Rip2 antibody followed by immunoblotting with an anti-ubiquitin antibody. Immunoprecipitates were also immunoblotted with a Rip2 antibody to insure that equal amounts of protein were immunoprecipitated.

Live intracellular Mycobacteria were required to stimulate the Nod-Rip2 pathway, indicating that the bacterium actively participated in this process, likely via the translocation of bacterial products into the cytosol. A specialized protein secretion system, encoded by the ESX1 locus, has been implicated in the perturbation of the host membranes [29],[30],[37] and for stimulation of the type I IFN response [28] and inflammasome activation [38], suggesting that this system might contribute to cytosolic recognition via Nod proteins. In order to test this hypothesis, we infected the mouse macrophage cell line RAW 264.7 with wild type Mtb or mutants lacking ESX1 function. No induction in Rip2 polyubiquitination was observed upon infection with a strain of Mtb harboring the “RD1” mutation, which deletes a portion of the ESX1 locus [39]. Similarly, a mutant lacking *espA*, a distally-encoded gene that is required for ESX1-mediated secretion [40], also failed to elicit this response (Figure 2). The phenotype of the latter mutant could be complemented by the expression of *espA* from a plasmid vector, demonstrating that the inability to stimulate Rip2 polyubiquitination was linked to the *espA* mutation. Furthermore, *M. bovis* BCG, an attenuated vaccine strain carrying the RD1 deletion and therefore lacking ESX1 function [39], was unable to stimulate Rip2 polyubiquitination. While all of these ESX1 mutants are less virulent than wild type bacteria, the lack of Nod2-Rip2 stimulation did not appear to be a nonspecific effect of attenuation. Two unrelated bacterial mutants that are unable to grow intracellularly, a biotin auxotroph (Δ*bioF* [41]) and a small molecule efflux mutant (TN::*rv1410c* [42]), robustly stimulated this response (Figure 2). Taken together, these observations indicate that a functional ESX1 secretion system is specifically required for Nod2 stimulation.

### Membrane damage allows Nod2-mediated recognition of ESX1 mutants

**Figure 3:**
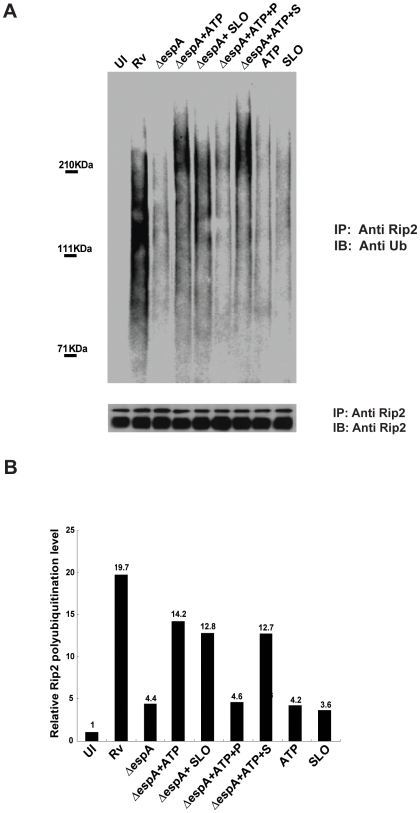
Membrane damage allows Nod2-mediated recognition of ESX1 mutants. A. The mouse RAW 264.7 macrophage cell line was was treated with ATP (5 mM) or SLO (5 µg/ml) for 15 minutes before infection with virulent wild type (Rv) or ESX1 mutant (Δ*espA*) strains of Mtb. After one hour, cell lysates were immunoprecipitated with a Rip2 antibody followed by immunoblotting with an anti-ubiquitin antibody. Immunoprecipitates were immunoblotted with a Rip2 antibody to insure that equal amounts of protein were immunoprecipitated. To verify that ATP was acting via the PANX1 protein, cells were pretreated with 500 µM of either a PANX1 blocking peptide (+P) or control scrambled peptide (+S) for 15 minutes prior to ATP addition. B. Relative abundance of Rip2 polyubiquitination in each sample as determined by densitometry of the results in panel A. All values were calculated relative to the uninfected sample (UI).

Since the Mtb-induced Rip2 polyubiquitination required ESX1, we hypothesized that this system might be responsible for the release of Nod2 ligands into the cytosol, perhaps via the disruption of vacuolar membrane integrity. However, it also remained possible that ESX1-deficient strains simply lacked a critical PAMP or other Nod2 stimulating activity. To distinguish between these possibilities, we investigated whether ESX1 function could be complemented by two exogenous membrane-disruptive activities. Streptolysin O (SLO) is a cholesterol-dependent toxin that introduces pores directly into mammalian membranes. Pores can also be introduced by adding ATP to macrophages, resulting in stimulation of the P2X7 receptor and the subsequent opening of the hemichannel, pannexin-1 (PANX1) [43]. We observed that membrane perturbation by either of these two methods resulted in robust Rip2 polyubiquitination upon infection with *espA*-deficient bacteria, which were otherwise unable to induce this response (Figure 3). The involvement of PANX1 in the ATP-facilitated Rip2 ubiquitination was verified by the addition of a competitive inhibitory peptide of the PANX1 pore. This peptide, but not a scrambled control peptide, inhibited Rip2 polyubiquitination to levels observed in cells infected with the Δ*espA* mutant (Figure 3). While the K^+^ flux subsequent to membrane damage has been found to stimulate NLRs in some circumstances [5], we found that the addition of ATP or SLO alone resulted in a minimal response. These data indicate that SLO, PANX1 and ESX1 are all likely to promote Nod2 pathway activation via a similar mechanism, by facilitating the release of a stimulatory mycobacterial component into the cytosol. Since this pathway appears to be specific for peptidoglycan fragments, mycobacterial MDP-containing fragments were the most likely candidates.

### The cytosolic Nod2-Rip2 pathway contributes to type I IFN production upon Mtb infection

**Figure 4:**
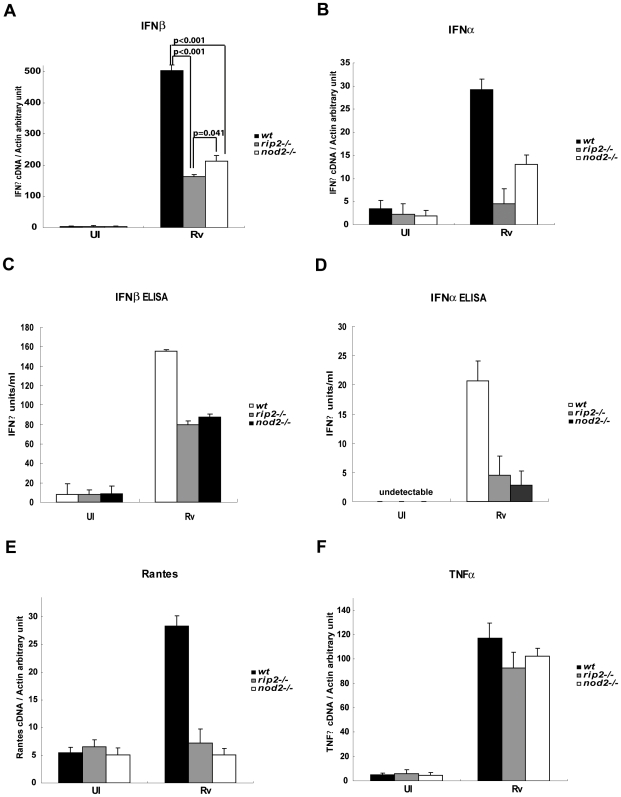
Type I Interferon production upon Mtb infection is reduced in Rip2- and Nod2-deficient macrophages. A, B, E, and F. BMDM derived from *wt*, *rip2−/−* and *nod2−/−* mice were infected with Mtb (MOI 10) for 4 h. RNA was harvested and IFNα, IFNβ, RANTES and TNFα mRNA levels were quantified using real time PCR. Gene expression is reported as copy number per 1,000 copies of β-actin. Samples were assayed in triplicate; error bars represent the standard deviation. The experiment shown is representative of at least three. Statistical evaluation was performed using an unpaired Student's t test. p-values>0.05 are reported as “n.s.” (*i.e.* not significant). C and D. BMDM derived from *wt*, *rip2−/−* and *nod2−/−* mice were infected with Mtb (MOI 10) for 18 h, the amount of IFNα and IFNβ released in the supernatant was quantified by ELISA. Samples were assayed in triplicate; error bars represent the standard deviation. N.D. indicates not detected, that is the actual value is below zero in standard curve.

The inability of ESX1 mutants to stimulate either the Nod2-Rip2 pathway or the type I IFN response [28] led us to hypothesize that the Nod2 pathway may mediate type I IFN expression in this system. To investigate a potential link between Nod2 and IFNα/β, we infected Nod2- or Rip2-deficient macrophages with Mtb, and measured the induction of IFNα and IFNβ mRNAs using real time PCR (qRT-PCR). In the absence of Rip2, IFNβ induction was reproducibly reduced approximately 3-fold, whereas IFNα induction was almost completely abrogated (Figure 4A and B). Nod2 deficiency had a similar effect on both IFNα and IFNβ transcription, consistent with its requirement for Rip2 polyubiquitination. Nod1 appears to play no role in this pathway, as *nod1−/−* macrophages produced wild type levels of IFNβ (Figure S1). The decreases in mRNA abundance observed in *rip2−/−* and *nod2−/−* cells were reflected in a similar decrease in protein production, as measured by ELISA (Figure 4C and D).

In order to assess the importance of Nod2 and Rip2 to the downstream IFNα/β-dependent macrophage response, we quantified the induction of RANTES mRNA, which depends on type I IFN secretion and signaling via the IFNαβ receptor (IFNAR1) in this infection model [24]. We found that in the absence of Rip2 or Nod2, Mtb infection failed to induce RANTES expression (Figure 4E). These data suggest that the effect of a Rip2 deficiency on downstream type I IFN responses may be even more pronounced than the IFNβ mRNA levels indicate. In contrast, TNFα mRNA levels were unaffected by Nod2- or Rip2-deficiency (Figure 4F) indicating that other pattern recognition pathways remained responsive to Mtb in these cells.

**Figure 5:**
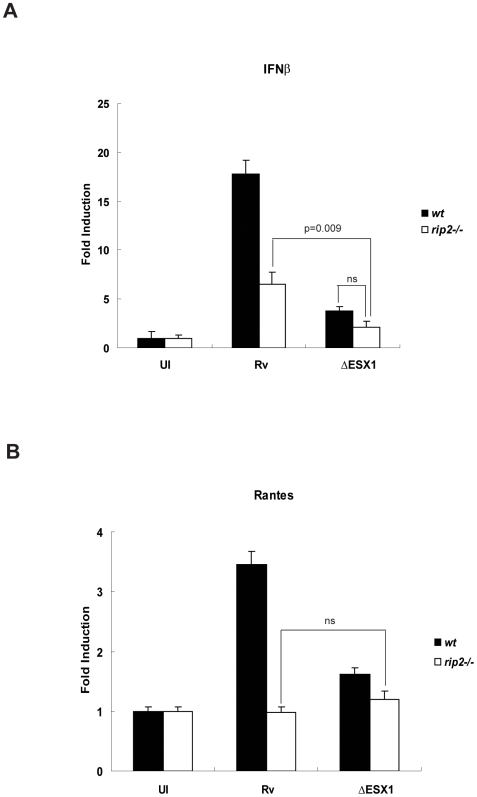
Multiple cytosolic pathways lead to IFNβ induction. A. BMDM derived from *wt*, *rip2−/−* and *nod2−/−* mice were infected with virulent Mtb H37Rv (Rv) or with an ESX1 mutant (ΔESX1) at an MOI of 10 for 4 h. RNA was harvested, and IFNβ mRNA levels were quantified using real time PCR. Gene expression of IFNβ is reported as copy number per 1,000 copies of β-actin. Samples were assayed in triplicate; error bars represent the standard deviation. The experiment shown is representative of at least three. B. BMDM derived from *wt*, *rip2−/−* and *nod2−/−* mice were infected with virulent Mtb H37Rv (Rv) or with an ESX1 mutant (ΔESX1) at an MOI of 10 for 4 h. RNA was harvested, and RANTES mRNA levels were quantified using real time PCR. Gene expression of RANTES is reported as copy number per 1,000 copies of β-actin. Samples were assayed in triplicate; error bars represent the standard deviation. The experiment shown is representative of at least three.

Consistent with previous work [28], we found that infection with ESX1 mutant bacteria induced significantly less IFNβ and RANTES expression than wild type bacteria (Figure 5). To test whether ESX1-mediated type I IFN expression was mediated solely via Rip2, we infected Rip2-deficient macrophages with ESX1 mutant bacteria and quantified IFNβ and RANTES mRNA levels. We found that in the absence of Rip2, the loss of ESX1 function resulted in a further decrease in IFNβ mRNA levels (Figure 5A), suggesting the presence of an additional host pathway(s) that contribute to IFNβ induction. However, Rip2 deletion had no significant effect in the absence of ESX1 (Figure 5B), supporting our biochemical evidence that NOD2 stimulation depends entirely the ESX1-dependent delivery of stimulants into the cytosol.

### The *N*-glycolylated MDP produced by Mtb is a potent stimulator of the Nod2-mediated type I IFN response

**Figure 6:**
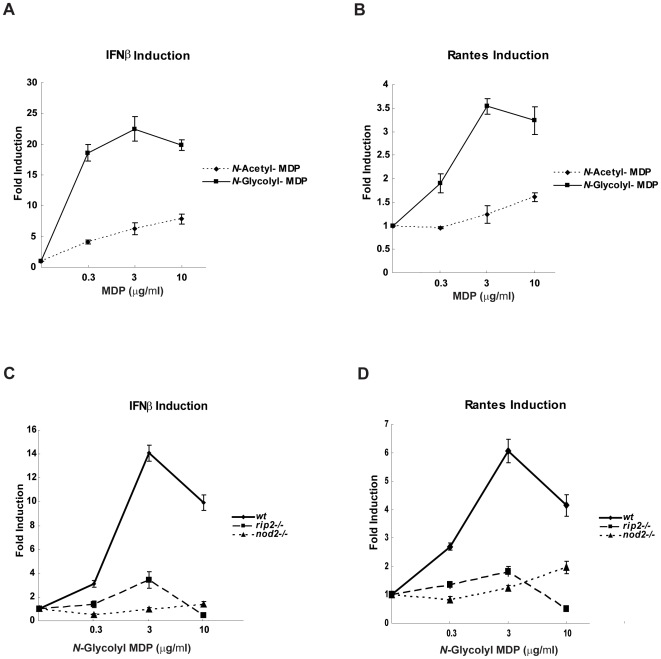
Nod2 stimulation is sufficient for type I IFN induction. A and B. *N*-Glycolyl MDP is more potent than the more common *N*-Acetylated derivative. The macrophage cell line RAW 264.7 was treated with the indicated concentrations of *N*-Glycolyl-MDP or *N*-Acetyl-MDP for 4 h. RNA was harvested, and IFNβ and RANTES mRNA levels were quantified using real time PCR. Gene expression of IFNβ (A) and RANTES (B) was normalized to β-actin then normalized to untreated control to estimate fold induction. Samples were assayed in triplicate; error bars represent the standard deviation. The experiment shown is representative of at least three. C and D. The *N*-Glycolyl-MDP-induced type I IFN response is Rip2- and Nod2- dependent. *Wt*, *rip2−/−* and *nod2−/−* transformed macrophage cell lines were treated for 4 hours with increasing concentrations of *N*-Glycolyl-MDP. RNA was harvested and IFNβ and RANTES mRNA levels were quantified using real time PCR. Gene expression of IFNβ (C) and RANTES (D) was normalized to β-actin and compare to untreated control to establish the fold induction. Samples were assayed in triplicate; error bars represent the standard deviation. The experiment shown is representative of at least three.

While our data indicated that a significant fraction of the IFNα/β response could be attributed to the Nod2-Rip2 pathway, it has been suggested that MDP stimulation alone is unable to induce type I IFNs and can only augment responses triggered by other pathways [36]. Indeed, we also found that the *N*-acetylated MDP that is commonly used to stimulate Nod2 was a very poor inducer of IFNβ and RANTES expression (Figure 6A and B). However, our preliminary studies investigating Rip2 polyubiquitination indicated that Mtb was a particularly potent stimulator of this response [21], and therefore we reasoned that this could be due to the *N*-glycolylated form of MDP produced by Mtb. To determine if this form of MDP was sufficient to induce type I IFN responses, we compared the ability of *N*-acetyl- and *N*-glycolyl-MDP to stimulate IFNβ expression. In contrast to *N*-acetyl MDP, treatment with the *N*-glycolylated form stimulated a robust IFNβ response, which was entirely dependent on Rip2 and Nod2 (Figure 6). In addition, at least 30-fold less *N*-glycolyl-MDP was necessary to stimulate the IFNβ transcription. Thus, the Nod2/Rip2 pathway alone is sufficient to induce the production of the IFN response when stimulated with this potent form of MDP.

### Induction of the host type I IFN response upon Mtb infection requires the Tbk1 kinase and Irf5

**Figure 7:**
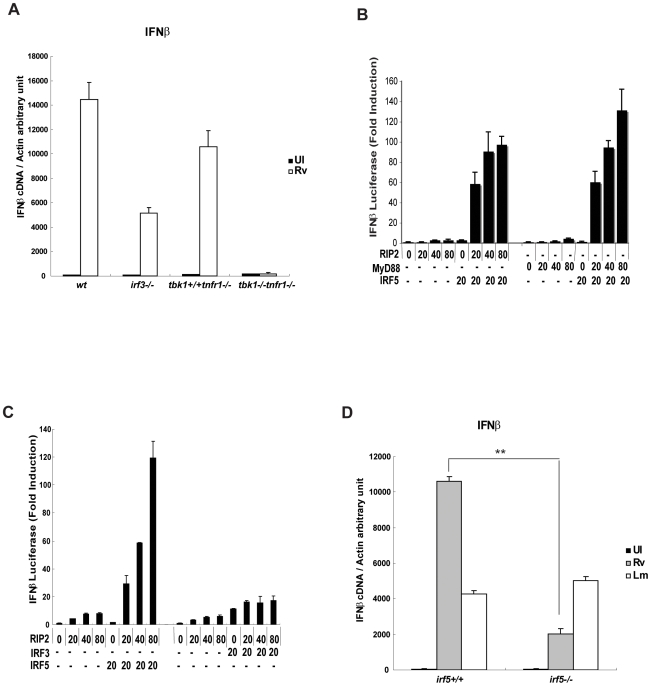
Mtb-induced type I IFN response is Tbk1-dependent and mediated through both Irf3 and Irf5. A. *M. tuberculosis*-induced type I IFN response is Tbk1-dependent and only partially mediated through Irf3. BMDM derived from *wt*, *irf3−/−* and *tbk1−/−tnfr1−/−* mice and littermate controls were infected with virulent Mtb H37Rv (Rv) at an MOI of 10 for 4 h. RNA was harvested and IFNβ mRNA level was quantified using real time PCR. Gene expression is reported as copy number per 1,000 copies of β-actin. Samples were assayed in triplicate; error bars represent the standard deviation. The experiment shown is representative of at least three. B. Co-expression of RIP2 and IRF5 stimulate IFNβ luciferase reporter activity. HEK293T cells were co-transfected with IFNβ-luciferase reporter plasmid (40 ng) together with the indicated concentrations of MyD88, IRF5 and RIP2 expression plasmids. Luciferase activity was measured 24 h later using Dual Luciferase reporter assay system (Promega). Renilla luciferase gene (40 ng) was co-transfected and used as an internal control. Each experiment was repeated three times. Data are expressed as mean±s.d. of three replicates. C. Co-expression of RIP2 and IRF3 does not stimulate IFNβ luciferase reporter activity. HEK293T cells were co-transfected with IFNβ-luciferase reporter plasmid (40 ng) together with the indicated concentrations of IRF5, IRF3 and RIP2 expression plasmids. Luciferase activity was measured 24 h later using Dual Luciferase reporter assay system (Promega). The Renilla luciferase gene (40 ng) was co-transfected and used as an internal control. Each experiment was repeated three times. Data are expressed as mean±s.d. of three replicates. D. Irf5 is required for an optimal type I IFN response upon Mtb infection. BMDM from *irf5−/−* or control littermates were infected with virulent Mtb H37Rv (Rv) at an MOI of 10, or with *Listeria monocytogenes* (Lm) strain 10403S (MOI 10) for 4 hours. RNA was harvested and IFNβ mRNA level was quantified by real time-PCR. IFNβ mRNA levels are reported as copy number per 1,000 copies of β-actin. Samples were assayed in triplicate; error bars represent standard deviation. Data shown is representative of at least three independent experiments.

*Listeria monocytogenes* infection induces a potent host type I IFN response mediated by the Tbk1 kinase and Irf3 [27],[32],[35],[44]. To test whether Mtb infection triggered similar pathways, we infected Irf3-deficient and Tbk1/Tnfr1-deficient macrophages with Mtb and measured IFN induction. The Tnfr1 deficiency was necessary to suppress the embryonic lethality of Tbk1 deletion [45]. Similar to the *L. monocytogenes* model, we found that IFNβ induction by Mtb infection was completely dependent upon Tbk1, and the loss of Tnfr1 had little effect (Figure 7A). However, in contrast to the complete dependence on Irf3 observed for *L. monocytogenes* [27],[32],[35], we found IFNβ expression was reduced, but not ablated when Irf3-deficient macrophages were infected with *M. tuberculosis* (Figure 7A). This partial dependence on Irf3 was not changed by varying the multiplicity of infection (Figure S2). These data prompted us to test whether other IRFs mediate Nod2-dependent type I IFN responses.

Induction of IFNβ expression is dependent on the formation of the enhancesome which includes the NF-κB, ATF-2, c-jun, Irf3 and Irf7 transcription factors [46]. Irf5 is a related family member that has also been shown to contribute to induction of type I IFN responses triggered by TLRs, and overexpression of MyD88 has been shown to synergize with Irf5 to induce IFNβ expression [47]. Based on these studies, we tested whether RIP2 collaborates with IRF5 or IRF3 to stimulate IFNβ luciferase reporter activity. HEK293 cells were transfected with an IFNβ-luciferase reporter construct, along with increasing amounts of expression plasmids encoding RIP2, MyD88, IRF3 or IRF5. RIP2 and IRF5 coexpression stimulated IFNβ promoter activity in a dose dependent manner and to a similar extent as MyD88 and IRF5 (Figure 7B). In contrast, RIP2 and IRF3 expression failed to induce this robust response (Figure 7C). RIP2 and IRF5 expression also stimulated IFNα4 promoter activity as well as a reporter construct containing multimerized ISRE elements (data not shown).

**Figure 8:**
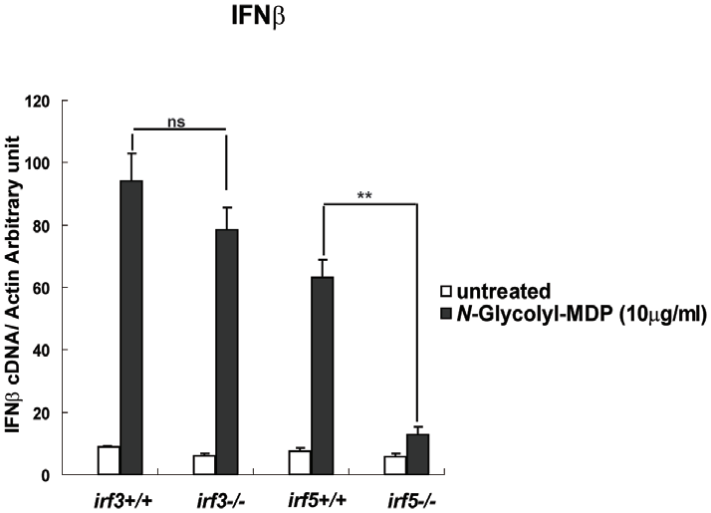
The *N*-Glycolyl-MDP-induced type I IFN response is Irf5-dependent and Irf3 independent. BMDM derived from *irf3−/−* and *irf5−/−* mice and their littermate controls were left untreated or treated for 6 hours with 10 µg/ml of *N*-Glycolyl-MDP. RNA was harvested, and IFNβ mRNA levels were quantified using real time PCR. IFNβ mRNA levels are reported as copy number per 1,000 copies of β-actin. Samples were assayed in triplicate; error bars represent the standard deviation.

To further investigate the contribution of Irf5 to the anti-bacterial type I IFN response, we infected macrophages from Irf5-deficient mice and control littermates with either Mtb or *L. monocytogenes*, and measured IFNβ expression. Consistent with the luciferase reporter studies, we found that Mtb-induced IFNβ (Figure 7D) and IFNα (Figure S3) expression was impaired in the absence of Irf5. In contrast, the response to *Listeria* was unaffected by the loss of Irf5 (Figure 7D). While the related Rip1 adaptor protein regulates Irf7 activity in innate anti-viral signaling [48], we found that IFNβ induction after Mtb infection was unaffected by Irf7 deficiency (data not shown). To rule out the possibility that Irf3 expression levels may also be affected in *irf5−/−* macrophages, we verified that the Irf3 protein level was unchanged in Irf5-deficient cells (Figure S4). These results indicated that Mtb infection stimulates type I IFN expression via a pathway that depends on Nod2, Rip2, Tbk1, and Irf5. This contrasts with the pathway triggered by *L. monocytogenes*, which depends entirely on Irf3 and not Irf5. We reasoned that this dependence on different Irf proteins might be explained by the preferential stimulation of a Nod2-Rip2-Irf5 pathway by mycobacterial peptidoglycan. Consistent with this model, we found that the IFNβ induction triggered by *N*-glycolyl MDP was entirely dependent on Irf5 and independent of Irf3 (Figure 8), functionally linking Irf5 with the Nod2 pathway.

## Discussion

Mammals first detect microbial infections via an array of PRRs that include both cell surface TLRs and cytosolic NLRs. However, not all microbial interactions represent a pathological state, and the immune system must be able to discriminate to some degree between colonization by commensal organisms and dangerous infection. One level of discrimination is provided by the desensitization or anatomical sequestration of TLRs at sites of chronic stimulation, such as the gut, which presumably allows for tolerance to normal flora [49],[50]. Bacterial pathogens can still be recognized at these sites via NLRs, since these systems rely on the specific ability of pathogens to translocate PAMPs into the host cytosol.

The concept that NLRs are specific for pathogenic organisms that disrupt host membranes is supported in a number of bacterial systems in which the loss of specific virulence functions abrogates NLR signaling. For example, in resting macrophages, cytosolic recognition of *L. monocytogenes* requires the pore-forming toxin, listeriolysin O [26],[27]. Similarly, *Helicobacter pylori* [51] and *Legionella pneumophila* [32] mutants lacking a functional type IV secretion system (T4SS), and *Shigella flexneri* [52] or *Salmonella enterica serovar typhimurium* [52] mutants lacking a functional type III secretion system (T3SS) fail to stimulate NLR pathways. In each case, the virulence system in question is responsible for host membrane damage and the likely translocation of bacterial products into the cytosol where they can be recognized by NLRs and/or other cytosolic surveillance systems.

Similarly, we found that the ESX1 specialized protein secretion system of Mtb is required for Nod2 recognition. While it has been suggested that type I IFN induction via ESX1 might represent a specific immunomodulatory virulence strategy [28], analogies to these other pathogens suggests that perhaps NLR recognition is simply a byproduct of a membrane damaging function that allows bacterial products to enter the cytosol. This model is supported by our observations that other membrane perturbing agents, such as SLO and PANX1 can substitute for ESX1 function and allow cytosolic recognition. Thus, in a number of cases it appears that NLRs can be considered as sentinels for pathogens that rely on membrane damage as a pathogenic strategy.

Based on their common role in protein secretion and in facilitating cytosolic recognition, it is tempting to speculate that ESX1 and Gram-negative T3SS and T4SS function analogously to deliver effector proteins into the host cytosol. Despite these similarities, the role played by ESX1 during infection remains unclear, since no translocated effectors have been identified to date. In both Mtb and *M. marinum*, a related pathogen of ectotherms, ESX1 has been implicated in host membrane disruption and one of the major substrates of this system, EsxA, has been proposed to possess a membrane-lytic activity [30],[37]. This single activity could be sufficient to account for the delivery of MDP and other PAMPs to the cytosol. It remains to be determined whether perturbing host membranes is the only role played by ESX1 during infection, or if this system also serves additional functions analogous to the specialized secretion systems of other pathogens.

A major consequence of the cytosolic recognition of Mtb is the induction of type I IFN. While the importance of this response in viral defense is clear and virtually universal, its role in antibacterial immunity appears to vary. Mice deficient in the type I IFN receptor, Ifnar1, are significantly more susceptible to several Gram-positive and -negative bacterial infections [53],[54],[55],[56], indicating that IFNα/β are important for immunity to many bacteria. However, Ifnar1 mutation has the opposite effect on the outcome of *L. monocytogenes* infection [57], suggesting that IFNα/β can also exacerbate disease. The role played by IFNα/β in Mtb infection remains somewhat uncertain. The induction of several immunologically important genes, including NOS2, depend on IFNα/β, suggesting a protective role. Initial studies of mouse and human infections appeared to support this view [58],[59]. However, like the *L. monocytogenes* system, mutation of the IFNα/β receptor has in most cases been associated with decreased bacterial burden in mouse models of tuberculosis [28],[59],[60],[61],[62]. IFNα/β may fail to protect against disease because Mtb inhibits the response to these cytokines in infected macrophages [63]. The ultimate influence of IFNα/β on Mtb infection appears to depend on a number of experimental factors, which might include host species, bacterial strain, route of infection and dose. Despite these differences however, some important themes emerge from these studies. Most importantly, the effect of IFNα/β is most apparent after the onset of adaptive immunity and not before, suggesting that the major role-played by type I IFNs during tuberculosis may be to instruct the priming or maintenance of the adaptive immune response and perhaps to control the differentiation of regulatory T cells [59].

A variety of bacterial pathogens trigger the type I IFN response, and a paradigm has begun to emerge regarding the induction of this response by bacteria. One current model suggests that bacterial DNA translocated into the host cytosol is the major eliciting agent. This model is based largely on the observations that infection with *L. monocytogenes* or *L. pneumophilla*, or transfection of DNA into the cytosol induces a similar IFNβ response that is Rip2 independent, and Tbk1- and Irf3-dependent [32]. Other PAMPs, such as MDP, can provide a synergistic IFN-inducing stimulus, but have not appeared to be sufficient for induction of IFNβ in the absence of other triggers [36].

In contrast, our data support a model whereby Nod2 stimulation by Mtb infection induces the polyubiquitination of Rip2, which acts via the Tbk1 kinase to stimulate the activity of Irf5 and induce transcription of IFNα/β. This differs from the pathway triggered by other bacteria such as *L. monocytogenes*, which depends entirely on Irf3 in resting macrophages [32] and does not involve Irf5 (Figure 7). Although Irf5 has previously been shown to be activated by the MyD88-dependent TLR7 and TLR9 pathways, this work reveals a novel role for this protein in Nod2 signaling, and a new link between Nod proteins and the type I IFN response. Furthermore, we found that unlike the *N*-acetylated MDP found in many bacteria, stimulation with the *N*-glycolylated MDP derivative found in mycobacteria was sufficient to stimulate the IFN response in the absence of other stimuli.

A significant component of IFNβ induction remains intact upon Mtb infection of Rip2-deficient macrophages (Figures 4 and 5), indicating that additional pathways are also involved. Since virtually all IFNβ expression is ESX1-dependent, it appears that the residual induction observed in *rip2−/−* macrophages also depends on cytosolic recognition pathways. These pathways could certainly include a DNA sensor that acts via Irf3, as proposed for other infections, since Irf3 deficiency had a moderate effect on IFNβ expression in our experiments (Figure 7A and S2). Thus, our data do not imply that Mtb is stimulating IFNα/β in a fundamentally different manner from other bacteria. Instead, it is likely that bacterial pathogens stimulate the IFN response via multiple, partially redundant pathways, and that the relative importance of each is determined by the unique biology of the infection. In the case of Mtb, we speculate that the *N*-glycolylation of its peptidoglycan, and perhaps a paucity of other stimulants such as DNA, favor recognition via Nod2. It is also possible that the balance of these pathways might be affected by the activation state of the macrophage. When resting macrophages are infected with *L. monocytogenes*, the IFN response requires LLO and is completely Irf3 dependent. In contrast, IFNγ-stimulated cells are able to deliver this bacterium to the lysosome, where the cell wall is degraded to produce abundant peptidoglycan fragments. In this situation, a significant component of the IFNβ induction depends on Nod2 and not Irf3 [64]. While Irf5 was not investigated in this study, it is possible that this represents another situation in which robust Nod2 signaling promotes a Nod2- and Irf5- dependent type I IFN response.

While we found that loss of Nod2-Rip2 signaling only partially reduces the induction of IFNβ, Rip2 deletion completely abrogated IFNα and RANTES expression. These results can be explained by the structure of the IFN regulatory circuit. Initially, only IFNβ is expressed, and subsequently IFNα and other interferon regulated genes (IRGs), such as RANTES, are induced via an Ifnar1 and Irf7-dependent autocrine/paracrine signaling pathway [65]. Thus, it appears that the decrease in IFNβ expression that we observe is sufficient to severely impair downstream IRG induction, at least in this cell culture model.

Multiple steps of this pathway are likely to depend on stable ubiquitin modifications. Not only did we observe that Rip2 is polyubiquitinated upon infection, but we also found that a Rip2 point mutant that cannot be stably ubiquitin modified is unable to mediate IFNα/β induction in response to Mtb infection (Figure S5). Collectively, these data suggest that polyubiquitinated Rip2 is required for Mtb-induced type I IFN expression via Irf5. Interestingly, MyD88-dependent activation of Irf5 involves formation of a tertiary complex that includes the E3 ubiquitin ligase, Traf6 [66],[67]. This E3 ubiquitin ligase associates with Rip2 upon MDP stimulation, raising the possibility that a Rip2-Traf6-Irf5 complex might exist and that the activity of Irf5 might also be regulated by ubiquitin.

The specificity of the innate immune system has been shaped by the very powerful natural selection imposed by microbial pathogens. Our work suggests that upon infection with Mtb, a particularly potent form of MDP is translocated into the host cell cytosol where it triggers a novel signaling pathway leading to the robust induction of the type I IFN response. It is unlikely to be coincidental that the active component of our most potent adjuvant, complete Freund's adjuvant (CFA), consists of mycobacterial cell fragments. The specific pathway described in this work might play a major role in this adjuvant's effectiveness, since IFNα/β production is required for CFA to promote antigen-specific immune responses (55). Thus, while PAMPs are often regarded as invariant microbial components, it is clear that functionally important pathogen-specific differences exist in the composition of these molecules, and that the immune system can differentiate these subtly distinct structures.

Given the potent adjuvant activity of mycobacterial components, it is somewhat surprising that the attenuated vaccine strain *M. bovis* BCG, which produces the same PAMPs present in CFA, provides poor protection against pulmonary TB in adults [68],[69]. The lack of ESX1 function in this strain appears to be at least partially responsible, since the reconstitution of ESX1 improves the efficacy of this vaccine [70],[71]. While this effect has previously been attributed to either the secretion of additional antigens or altered antigen presentation, it is also possible that ESX1 activity improves immunity by delivering crucial PAMPs into the cytosol where they are optimally recognized. Understanding both the details of PAMP trafficking, as well as the precise specificity of PAMP recognition, promises to aid in both the design of improved adjuvants and more effective tuberculosis vaccines.

## Materials and Methods

### Mice

C57BL/6 mice ages 8–12 weeks were obtained from the Jackson Laboratory. *rip2−/−* mice were a kind gift from Dr. Vishva M. Dixit (Genentech, Inc. South San Francisco, CA). *nod2−/−* mice were provided by Dr. Peter J. Murray (Department of Infectious Diseases, St. Jude Children's Research Hospital, Memphis, TN). *nod1−/−* and *nod1−/−nod2−/−* mice were provided by Dr. Gabriel Nunez (University of Michigan Medical School, Ann Arbor, MI). *irf3−/−*, *irf5−/−*, *tbk1+/+tnfr1−/−* and *tbk1−/−tnfr1−/−* mice and their littermate controls were provided by Dr. Kate A. Fitzgerald (University of Massachusetts Medical School, Worcester, MA). Mice were housed under specific pathogen-free conditions, and in accordance with the University of Massachusetts Medical School, IACUC guidelines.

### Bacteria

The WT strain of *M. tuberculosis* used in these studies was the H37Rv strain. All the mutants were derived from the wild type strain. ΔESX-1 was obtained from D. Sherman (SBRI, Seattle.WA) [39]. ΔBioF, ΔRv3616 and ΔRv3616-complemented strains have been described previously [40],[41]. TN::Rv1410 contains a himar-1 transposon inserted at nucleotide #688 of the 1557 bp predicted open reading frame [72]. All strains were cultured in 7H9 medium containing 0.05% Tween 80 and OADC enrichment (Becton Dickinson). Pre-titered stocks of *Listeria monocytogenes* strain 10403 stored at −80°C (kindly provided by Victor Boyartchuk) were recovered for 1 hr at 37°C in 9 ml of Tryptic Soy Broth (BD Biosciences). Bacteria were then washed and resuspended in PBS prior to infection.

### Antibodies and reagents

Anti-Rip2 (Rabbit) and anti-ubiquitin (Mouse) antibodies were obtained from Santa Cruz Biotechnology. Anti-Irf3 antibody was obtained from Zymed. Anti-Irf5 antibody was obtained from Abcam. Anti-β-actin antibody was obtained from Sigma. MDP was obtained from InvivoGen. Mouse TNF-α was obtained from Sigma. LPS derived from *Escherichia coli* strain 0111.B4 was purchased from Sigma, dissolved, treated with deoxycholate, and re-extracted with phenol/chloroform as described in [73]. The pannexin-1 mimetic blocking peptides panx1 (WRQAAFVDSY) and the scrambled peptide control were synthesized by GeneScript Corporation (Piscataway, NJ) and have been described previously [74]. Streptolysin O (SLO) a pore forming protein derived from *Streptococcus* and Adenosine 5′- triphosphate (ATP) were purchased from Sigma. *N*-glycolyl muramyl dipeptide (*N*-glycolyl MDP) was custom synthesized (Carbohydrate Synthesis, Oxford, UK) and shown to be more than 95% pure by NMR spectrometry. This preparation was found to be free of endotoxin contamination using the Limulus amebocyte lysate assay (Pyrotell, Cape Cod Inc., MA).

### Macrophage infections

Bone marrow from 8- to 10-week-old mice was harvested from femurs and differentiated into macrophages for 7 days in Dulbecco's modified Eagle medium (DMEM) supplemented with 10% L929-cell conditioned medium, 10% fetal bovine serum, 2 mM L-glutamine and 1 mM sodium pyruvate. After 7 days in culture, bone marrow derived macrophages (BMDMs) were washed with phosphate-buffered saline (PBS) and seeded into tissue culture plates for infection. RAW 264.7 macrophage cell line was cultured in Dulbecco's modified Eagle medium (DMEM) supplemented with 10% fetal bovine serum. All Mtb strains were cultivated in 7H9 broth, grown to exponential phase and washed thoroughly in DMEM media prior to infection. Bacterial clumps were removed by passing the washed suspension through a 5 µm syringe filter. For the peptide blocking studies, the cells were pre incubated with the desired peptides for 30 minutes followed by ATP or SLO for additional 15 minutes. Macrophages were infected at an MOI of 10 for 1 or 2 hours after which filtered cell lysates were immunoprecipitated with anti-Rip2 antibody (Santa Cruz). Heat inactivation was achieved by incubating the bacteria at 80°C for 30 minutes. Immortalized macrophage cell lines from wild type, *rip2−/−*, *nod2−/−* and *nod1−/−nod2−/−* mice were established by infecting bone marrow cells with a *v-raf/mil* and *v-myc* retrovirus in the presence of GM-CSF and polybrene [75],[76]. These *rip2−/−*, *nod2−/−* and *nod1−/−nod2−/−* macrophage cell lines express CD11b and Gr-1 and are capable of phagocytosing antibody coated beads. To determine the effect of cytochalasin D on the phagocytic function of the macrophages, we used the Vybrant phagocytosis assay kit to quantify the uptake of fluorescent *E. coli*. This assay was performed according to the protocol provided by the manufacturer.

### Immunoprecipitation and Western blot analysis

For the immunoprecipitation and ubiquitination assays, cell lysates were prepared in radioimmune precipitation assay (RIPA) buffer (150 mM NaCl, 50 mM Tris-HCl (pH 7.5), 1% NP40, 0.25% deoxycholate, 0.1% SDS, 1 mM EDTA), supplemented with protease inhibitors (Roche Applied Science) and 5 mM N-Ethylmaleimide (Sigma), immunoprecipitated with anti-Rip2 antibody (Santa Cruz). Polyubiquitinated Rip2 proteins were detected by immunoblotting with an anti-ubiquitin antibody (Santa Cruz). Total immunoprecipitated Rip2 protein was measured by immunoblotting with anti-Rip2 antibodies (Santa Cruz).

### Luciferase reporter assay

HEK293 cells (2×10^4^) seeded in 96 well plates were transfected with 40 ng of the IFNβ luciferase reporter plasmid together with a total of 100 ng of various expression plasmids using GeneJuice (Novagen). The total amounts of transfected DNA were kept constant in all experiments by adjustment with empty vector. Luciferase activity was measured 24 h later using Dual Luciferase reporter assay system (Promega). The *Renilla* luciferase gene (40 ng) was co-transfected and used as an internal control plasmid. IFNβ luciferase reporter activity was normalized to *Renilla* luciferase reporter activity. Each experiment was repeated three times. Data are expressed as mean±s.d. of three replicates.

### Real time quantitative PCR analysis

To measure IFNα/β mRNA levels upon MDP treatment or Mtb infection, total RNA was extracted from the macrophage cultures using Trizol reagent (Invitrogen) according to the manufacturer's directions. cDNA was prepared from 2 µg of total RNA and quantitative real-time PCR performed using SYBR green as a label with the following primers: mIFNα-F, 5′-AAGATGCCCTGCTGGCTG; mIFNα-R, 5′-TTCTGCTCTGACCACCTCCC; mIFNβ-F, 5′-CGTCTCCTGGATGAACTCCAC; mIFNβ-R, TGAGGACATCTCCCACGTCA; β-actin-F, 5′-CGAGGCCCAGAGCAAGAGAG; β-actin-R, 5′-CGGTTGGCCTTAGGGTTCAG; mTNFα-F, CAGTTCTATGGCCCAGACCCT; mTNFα-R, CGGACTCCGCAAAGTCTAAG; mRANTES-F, GCCCACGTCAAGGAGTATTTCTA; mRANTES-R, ACACACTTGGCGGTTCCTTC. Results shown are representative of more than three separate infection experiments, with each PCR performed in triplicate. All values reported were in the linear range of the experiment and were normalized to β-actin values. Standard curves were generated by linear dilution of a cDNA sample generated from poly I∶C-stimulated macrophages.

### ELISA

IFNα protein in cell culture supernatants was performed using a custom ELISA as described previously [77]. IFNα concentrations were calculated using a recombinant IFNα (HyCult, Biotechnology, Uden, Netherlands) standard curve performed in quadruplicate using linear regression, and expressed in units per ml. IFNβ protein in cell culture supernatants was measured similarly using a custom ELISA as described in [78].

## Supporting Information

Figure S1 Type I Interferon production upon Mtb infection is reduced in Rip2- and Nod2-deficient macrophages but not in Nod1-deficient macrophages. BMDM derived from *wt*, *nod1−/−*, *rip2−/−* and *nod2−/−* mice were infected with Mtb (MOI 10) for 4 h. RNA was harvested, and IFNβ mRNA levels were quantified using real time PCR. Gene expression is reported as copy number per 1,000 copies of β-actin. Samples were assayed in triplicate; error bars represent the standard deviation.
(0.23 MB PDF)

Figure S2 Mtb-induced type I IFN response is only partially mediated through Irf3. BMDM derived from *wt* and *irf3−/−* mice were infected with virulent Mtb H37Rv (Rv) at an MOI of 1, 3 and 10 for 4 h. RNA was harvested, and IFNβ mRNA level was quantified using real time PCR. Gene expression is reported as copy number per 10,000 copies of β-actin. Samples were assayed in triplicate; error bars represent the standard deviation.
(0.23 MB PDF)

Figure S3 Irf5 is required for an optimal type I IFNα response upon Mtb infection. BMDM from *irf5−/−* or control littermates were infected with virulent Mtb H37Rv (Rv) at an MOI of 10, or with *Listeria monocytogenes* (Lm) strain 10403S (MOI 10) for 4 hours. RNA was harvested, and IFNα mRNA level was quantified by real time-PCR. Gene expression of IFNα is reported as copy number per 1,000 copies of β-actin. Samples were assayed in triplicate; error bars represent standard deviation.
(0.23 MB PDF)

Figure S4 Irf3 and Irf5 expression levels in *irf3−/−* and *irf5−/−* macrophages. BMDM derived from *irf3−/−* and *irf5−/−* mice and their littermate controls were lysed in RIPA buffer and the Irf3 and Irf5 expression levels was determined by immunoblotting of anti Irf3 (Zymed) and Irf5 (Abcam) antibodies. Protein loading level was measured by β-actin antibody (Sigma).
(0.40 MB PDF)

Figure S5 Rip2 polyubiquitination is required for the Mtb-induced Type I IFN response. The *rip2−/−* transformed macrophage cell line was infected with the retroviral vector alone or with retroviruses expressing wild type Rip2 or a form of Rip2 (K209R) that cannot be ubiquitin modified [22]. The *rip2−/−* reconstituted macrophage cell lines were then infected with Mtb (MOI 10) for 4 h. RNA was harvested, and IFNβ mRNA levels were quantified using real time PCR. Gene expression is reported as copy number per 1,000 copies of β-actin. Samples were assayed in triplicate; error bars represent the standard deviation. Rip2 expression levels in each of the *rip2−/−* reconstituted macrophage cell lines were examined by immunoblotting to insure that equivalent expression levels of Rip2 were achieved.
(0.23 MB PDF)
