# Supplementary figures and images for: NOD2, RIP2 and IRF5 Play a Critical Role in the Type I Interferon Response to Mycobacterium tuberculosis
Source: PLoS Pathog. 2009 Jul 3;5(7):e1000500. doi: 10.1371/journal.ppat.1000500 (PMC2698121; doi:10.1371/journal.ppat.1000500)

Fig. S1

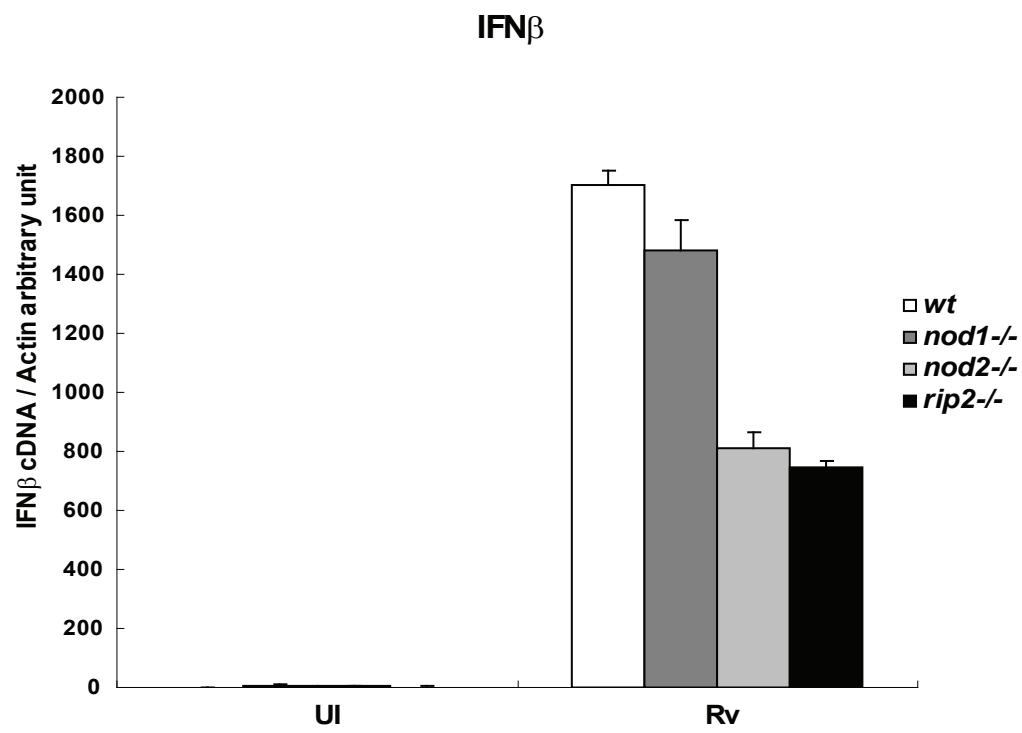

Supplement: Figure S1 — Type I Interferon production upon Mtb infection is reduced in Rip2- and Nod2-deficient macrophages but not in Nod1-deficient macrophages. BMDM derived from wt, nod1−/−, rip2−/− and nod2−/− mice were infected with Mtb (MOI 10) for 4 h. RNA was harvested, and IFNβ mRNA levels were quantified using real time PCR. Gene expression is reported as copy number per 1,000 copies of β-actin. Samples were assayed in triplicate; error bars represent the standard deviation. (0.23 MB PDF) [file ppat.1000500.s001.pdf]

Fig. S2

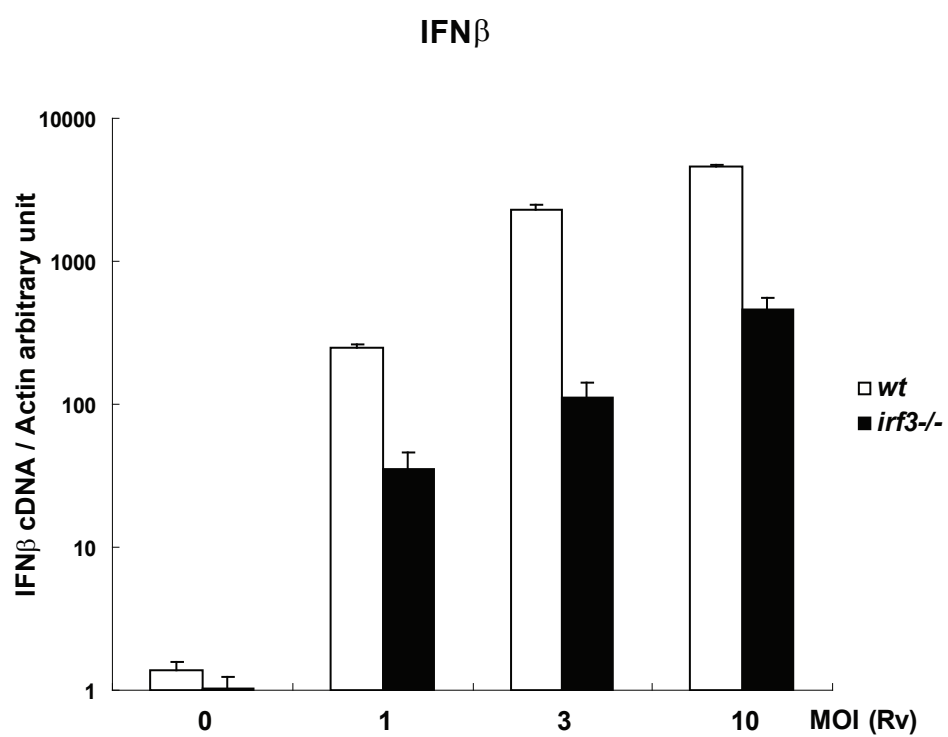

Supplement: Figure S2 — Mtb-induced type I IFN response is only partially mediated through Irf3. BMDM derived from wt and irf3−/− mice were infected with virulent Mtb H37Rv (Rv) at an MOI of 1, 3 and 10 for 4 h. RNA was harvested, and IFNβ mRNA level was quantified using real time PCR. Gene expression is reported as copy number per 10,000 copies of β-actin. Samples were assayed in triplicate; error bars represent the standard deviation. (0.23 MB PDF) [file ppat.1000500.s002.pdf]

Fig. S3

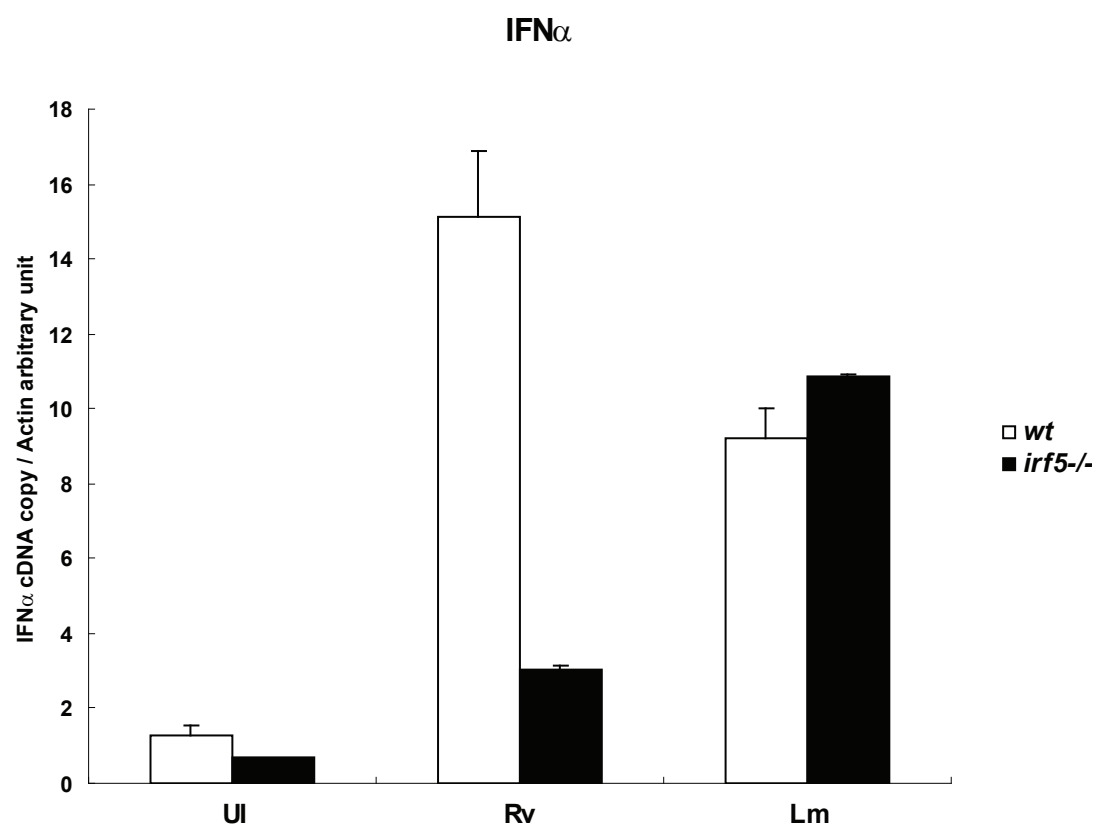

Supplement: Figure S3 — Irf5 is required for an optimal type I IFNα response upon Mtb infection. BMDM from irf5−/− or control littermates were infected with virulent Mtb H37Rv (Rv) at an MOI of 10, or with Listeria monocytogenes (Lm) strain 10403S (MOI 10) for 4 hours. RNA was harvested, and IFNα mRNA level was quantified by real time-PCR. Gene expression of IFNα is reported as copy number per 1,000 copies of β-actin. Samples were assayed in triplicate; error bars represent standard deviation. (0.23 MB PDF) [file ppat.1000500.s003.pdf]

**Fig. S4**

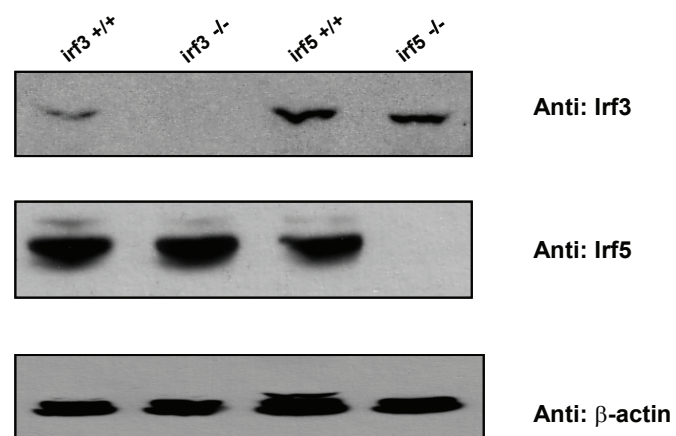

Supplement: Figure S4 — Irf3 and Irf5 expression levels in irf3−/− and irf5−/− macrophages. BMDM derived from irf3−/− and irf5−/− mice and their littermate controls were lysed in RIPA buffer and the Irf3 and Irf5 expression levels was determined by immunoblotting of anti Irf3 (Zymed) and Irf5 (Abcam) antibodies. Protein loading level was measured by β-actin antibody (Sigma). (0.40 MB PDF) [file ppat.1000500.s004.pdf]

Fig. S5

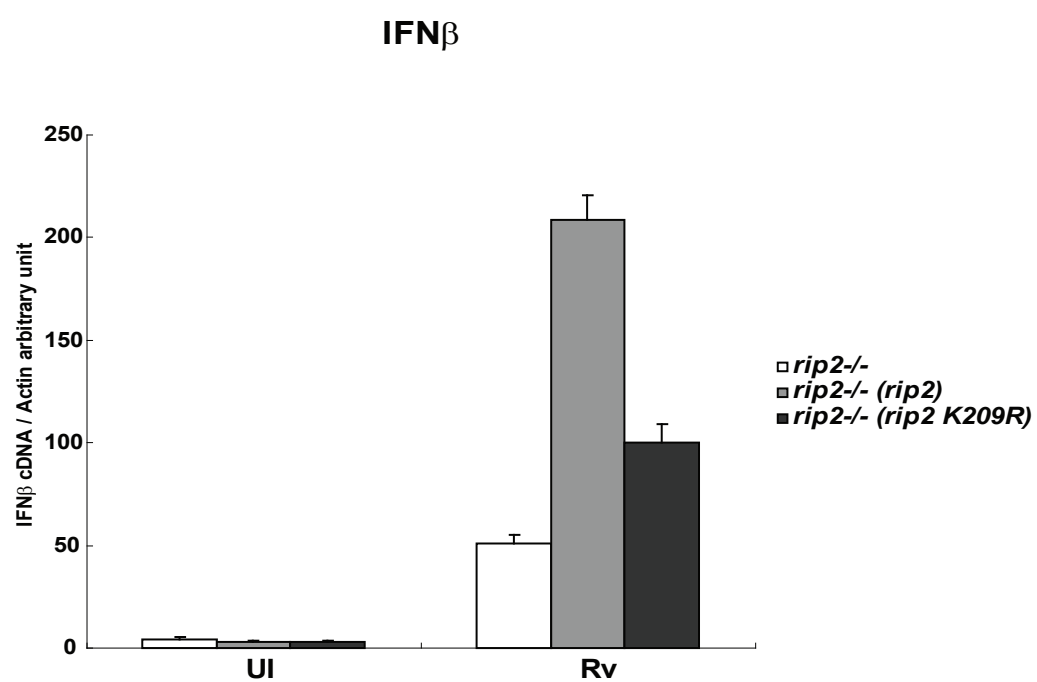

Supplement: Figure S5 — Rip2 polyubiquitination is required for the Mtb-induced Type I IFN response. The rip2−/− transformed macrophage cell line was infected with the retroviral vector alone or with retroviruses expressing wild type Rip2 or a form of Rip2 (K209R) that cannot be ubiquitin modified [22]. The rip2−/− reconstituted macrophage cell lines were then infected with Mtb (MOI 10) for 4 h. RNA was harvested, and IFNβ mRNA levels were quantified using real time PCR. Gene expression is reported as copy number per 1,000 copies of β-actin. Samples were assayed in triplicate; error bars represent the standard deviation. Rip2 expression levels in each of the rip2−/− reconstituted macrophage cell lines were examined by immunoblotting to insure that equivalent expression levels of Rip2 were achieved. (0.23 MB PDF) [file ppat.1000500.s005.pdf]
